# Localized Non-melanoma Skin Cancer: Risk Factors of Post-surgical Relapse and Role of Postoperative Radiotherapy

**DOI:** 10.1007/s11864-020-00792-2

**Published:** 2020-10-09

**Authors:** Francesca Caparrotti, Idriss Troussier, Abdirahman Ali, Thomas Zilli

**Affiliations:** 1 Department of Radiation Oncology, Geneva University Hospital, CH-1211 Geneva 14, Switzerland; 2 Faculty of Medicine, Geneva University, Geneva, Switzerland

**Keywords:** Radiotherapy, Postoperative, Adjuvant, Non-melanoma skin cancer, Basal cell carcinoma, Squamous cell carcinoma

## Abstract

The mainstay treatment of localized non-melanoma skin cancer (NMSC) is surgical excision or Mohs surgery. However, approximately 5% of patients with NMSC harbor high-risk clinicopathologic features for loco-regional recurrence, and distant metastasis. Prognostic factors such as close or positive margins, tumor size ≥ 2 cm, poor tumor differentiation, perineural invasion, depth of invasion, and immunosuppression have all been associated with increased loco-regional recurrence and impaired survival rates. In these patients more aggressive treatments are needed and radiotherapy (RT) is often discussed as adjuvant therapy after surgical resection. Due to the retrospective setting and the heterogeneity of the available studies, indications for adjuvant RT in patients with localized resected NMSC harboring high-risk features remain debated. Studies highlighting the limitations of our current understanding of the independent prognosis of each risk factor are needed to better define the role of adjuvant RT on outcome of localized NMSC and standardize its indications in the clinical setting.

## Introduction

Non-melanoma skin cancer (NMSC) is the most common type of neoplasia [1], with an incidence over 5 million cases annually in United States [2]. More than 95% of all NMSC are represented by basal cell carcinoma (BCC) and cutaneous squamous cell carcinoma (cSCC).

In contrast with melanoma which develops from melanocytes and represents a much more aggressive disease, NMSC develops from the keratinocytes of the skin and, in the majority of cases, has a better cure rate, mainly due to the fact that it remains limited to its primary site of disease. Most lesions (90%) appear in sun exposed regions, namely in the head and the neck, with only a minority (< 5%) that metastasizes to regional lymph nodes [3, 4], and are most commonly diagnosed in elderly people. However, in recent years, an increase in NMSC at younger ages has been described, due to prolonged unprotected sun exposure, the use of tanning beds, and an increase of immunosuppression [5].

The mainstay treatment of NMSC is surgical excision or Mohs surgery for the majority of patients especially those presenting a low-risk for relapse [6]. However, approximately 5% of patients with NMSC harbor high-risk clinicopathologic features for loco-regional recurrence, distant metastasis, and death [7, 8]. The American Joint Committee of Cancer (AJCC) describes several factors associated with high-risk of tumor recurrence and metastasis such as the site of disease (ears and lips), poor histological differentiation, perineural invasion (PNI), and host factors such as immunosuppression [9].

In patients with high-risk features more aggressive treatments are needed and radiotherapy (RT) is often discussed in the postoperative setting after surgical resection. Recently an American Society for Radiation Oncology (ASTRO) task force published guidelines on the role of RT in both definitive and adjuvant setting for NMSC (Table 1) [10••], gaining, however, negative critics from the American Academy of Dermatology for the low quality of evidence [12]. Indeed, the impact of major prognostic risk factors of post-surgical relapse and the role of postoperative RT for BCC and cSCC suffers from lack of high-level evidence, due to the paucity of prospective trials and well-defined practice guidelines.

**Table 1:** Clinical and pathological risk factors used for postoperative radiotherapy

ASTRO [10••]	Karia et al. [11]
Clinical or radiological PNI	PNI (≥ 0.1 mm nerves)
Close or positive margins with no possibility for further S	Size ≥ 2 cm
Recurrent tumors after a prior margin-negative resection	Poor tumor differentiation
T3–T4 tumors	Tumor invasion beyond fat
Desmoplastic or infiltrative cSCC in the setting of CI	

*Abbreviations*: *ASTRO*, American Society for Radiation Oncology; *PNI*, perineural invasion; *S*, surgery; *cSCC*, cutaneous squamous cell carcinoma; *CI*, chronic immunosuppression

In consideration of the limited consensus on this subject, the aim of this narrative review is to investigate on major prognostic factors of localized NMSC and shed light on the role of postoperative RT for patients with BCC and cSCC.

## Risk factors

Identification of risk factors is essential to define the prognosis and the most appropriate treatment approach.

The National Comprehensive Cancer Network (NCCN) guidelines describe several factors to classify a high-risk NMSC (Table 2) [13•]. All these factors influence the prognosis of the disease, but not all of them constitute an indication for adjuvant RT.

**Table 2:** High-risk factors for recurrence based on the NCCN version 1.2020 guidelines [6]

BCC	cSCC
Area L^1^ ≥ 20 mm	Area L^1^ ≥ 20 mm
Area M^2^ ≥ 10 mm	Area M^2^ ≥ 10 mm
Area H^3^	Area H^3^
Poorly defined borders	Poorly defined borders
Recurrent disease	Recurrent disease
Immunosuppression	Immunosuppression
Prior RT	Prior RT or chronic inflammatory process
Aggressive subtype^4^	Aggressive subtype^5^
PNI	PNI
	Lymphatic involvement
	Vascular involvement
	Neurologic symptoms
	Poorly differentiated tumor
	Rapidly growing tumor
	DOI ≥ 6 mm or invasion beyond subcutaneous fat

*Abbreviations*: *NCCN*, National Comprehensive Cancer Network; *BCC*, basal cell carcinoma; *cSCC*, cutaneous squamous cell carcinoma; *RT*, radiotherapy; *PNI*, perineural invasion; *DOI*, depth of invasion

^1^Trunk and extremities (excluding hands, nail units, pretibia, ankles, and feet)

^2^Cheeks, forehead, scalp, neck, and pretibia

^3^“Mask areas” (central face, eyelids, eyebrows, periorbital, nose, lips, chin mandible, preauricular, temple), genitalia, hands, feet

^4^Infiltrative, micronodular, morpheaform, basosquamous, sclerosing, carcinosarcomatous (mixed and in any portion of the tumor)

^5^Acantholytic (adenoid), adenosquamous, desmoplastic, metaplastic (carcinosarcomatous)

A review of 1818 cSCC cases aiming to compare different staging systems such as American Joint Committee on Cancer (AJCC), Union for International Cancer Control (UICC), and Birmingham Women’s Hospital (BWH), identified different risk factors that may require adjuvant RT (Table 1) [11]. The 10-year local recurrence rates are: 0 factors = 0.6%, 1 factor = 5%, 2–3 factors = 21%, and 4 factors or bone invasion = 67%.

Here below we detail high-risk factors abundantly described in literature such as surgical margin, PNI, histologic subtypes and tumor differentiation, tumor size, depth of invasion, and immunosuppression.

### Surgical margins

The large majority of NMSC are successfully treated with surgical resection, either with radical surgery or Mohs micrographic surgery. In both cases, the risk of achieving an incomplete or close margin exists, for example, on the face where clear margins are sometimes difficult to achieve.

A large retrospective series of patients treated with incomplete resection of BCC mainly located in the head and neck region, at the Princess Margaret Cancer Center, compared outcome between those receiving further treatment with adjuvant RT and those followed clinically [14]. The former group had both 5- ad 10-year relapse-free rates of 91%, while the latter had significantly lower relapse-free rates of 61% and 40% at 5 and 10 years, respectively. Moreover, they found that the 5-year local recurrence was 17% if the lateral margin was positive and 33% when both lateral and deep margins were involved. However, when analyzing the 10-year actuarial probability of local control for the lesions immediately treated with adjuvant RT and those initially observed, the authors found that their outcome was very similar (92% and 90%, respectively). This results from an excellent salvage rate by either RT or surgery at the time of failure in the clinically observed group of patients.

As for resected cSCC, localized on the lip, Babington et al. reported a local and/or regional relapse in 53% of patients treated exclusively with surgery, of whom 1/3 had either incomplete or close margins [15]. Based on their findings, a close margin harboring a high-risk of local failure was <2 mm. Finally, the group of patients treated initially with surgery only had an excellent outcome after salvage treatments with both surgery and RT, resulting in a 2-year overall survival of 100%.

Staub et al. assessed 844 patients with NMSC (80% BCC and 20% cSCC) after surgical excision and concluded that a 4 mm margin for BCC, 8 mm margin for morpheaform BCC, and 10 mm margin for cSCC lead to a 5-year recurrence rate inferior to 5% [16].

### Perineural invasion

NMSC with PNI have a higher incidence of recurrence and a poorer prognosis, harboring moreover the potential to spread directly into and beyond the skull base via the cranial nerves. When PNI is revealed through histology (pPNI) patients’ outcome is superior compared with cases in which PNI is clinically present (cPNI). As a matter of fact, according to a retrospective review of 118 cases of NMSC all treated with RT, Jackson et al. identified a “low-risk” group, consisting in BCC with pPNI, that compared with an “intermediate-risk” group of cSCC with pPNI, had a 5-year local control of 97% vs. 84%, respectively (*p* = 0.002) [17]. The “intermediate-risk” group was also found to have a 55% risk of regional relapse. The “high-risk” group consisted in tumors with cPNI with an estimated relapse-free rates of 46% at 5 years.

Another retrospective study on NMSC treated with RT or surgery, reported that extensive pPNI had worse outcome than focal pPNI (*p* = 0.008) but found no difference in outcome between pPNI involving nerves with a size of ≥ 0.1 mm vs. < 0.1 mm [18]. This latter finding was on the contrary found to be significantly correlated with outcome in another smaller retrospective cohort, with PNI ≥ 0.1 mm having worse local control, nodal recurrence and overall survival [19].

However, PNI remains often under reported, and clinicians need to know multiple features on pPNI, which include whether the PNI is intratumoral or extratumoral, if it extends beyond the dermis, what the distance to the nearest margin is, the size of the involved nerve/s, and finally if it is focal or multifocal. Finally, PNI is commonly associated with other risk factors, such as tumor size >2 cm, recurrent tumor, and poorly differentiated histology [20].

### Histology

Histopathological features of NMSC harbor prognostic information. BCC has generally a much better prognosis than cSCC due to the local growth pattern and the minimal risk of metastasis. Even in the presence of pPNI, BCC has a better 5-year loco-regional control than patients with cSCC (97% vs. 84%, *p* = 0.02) [17].

Different subtypes of BCC and cSCC exist, and some of them are more aggressive and carry a worse prognosis (Table 2), such as the desmoplastic cSCC subtype that is known to be a high-risk factor for local recurrence. Brantsch et al. reported that less than 2% of patients with non-desmoplastic cSCC ≤ 6 mm in thickness would experience local recurrence at 6 years, compared with about 25% of patients presenting desmoplastic cSCC or cSCC with tumor thickness > 6 mm (*p* < 0.0001) [21]. As for BCC, infiltrative and micronodular subtypes were found to be adverse factors significantly associated with local recurrence among a cohort of more than 300 patients with excised facial BCC [22].

In addition to the histologic subtypes, tumor differentiation also plays a role in disease recurrence. Poor histological differentiation is associated with the presence of PNI ≥ 1 mm diameter, and worse outcome [19, 23].

### Tumor size

Tumor size is an important prognostic factor and it is used to classify tumors in the TNM staging system.

Brantsch et al. prospectively assessed different risk factors for local recurrence and metastasis in 615 cSCC patients with a median follow-up of 43 months [21]. They found that tumors greater than 2 cm are associated with a significant risk of local recurrence independently of other risk factors (*p* < 0.0001). Also, patients with a tumor ≥ 6 cm have a 16% risk of developing metastasis. Others report the same worse prognosis for cSCC ≥ 2 cm [19, 24]. Although Veness et al. also found that size above 2 cm was a high-risk factor, they reported that size alone does not constitute an independent predictor of impaired outcome, as other factors (depth of invasion and thickness) need to be considered [25].

For BCC, not only size, but also location has been found to constitute a high-risk factor for recurrence. Silverman et al. reviewed 5755 cases of BCC and concluded that tumors located in high-risk zones such as the face area, presented more recurrences if they were greater than 6 mm, and tumors greater than 10 mm presented a higher risk of recurrence in any other skin region [26].

### Depth of invasion

Depth of invasion is described in literature mainly in tumor thickness (Breslow) given as a numeric variable in mm, or by tumor extension to different anatomic planes.

Ross et al. report that cSCC with a depth of invasion ≥ 10 mm was associated with lower disease-specific survival and overall survival (*p* = 0.004 and *p* = 0.08, respectively), and was also significantly associated with PNI of large diameter nerves (*p* < 0.001) [19]. With a cut-off of 7 mm, depth of invasion was not found to be correlated to worse disease-specific survival; however, invasion beyond the subcutaneous tissue was a relevant prognostic factor for disease-specific survival, in cSCC (*p* = 0.009) [27]. A large review including more than 20,000 cases of cSCC, found that tumor depth > 2 mm and > 6 mm were associated with the highest risk ratio of local recurrence and metastasis [24]. Finally, Jackson et al. found that patients presenting cSCC with muscle invasion had a worse 5-year recurrence-free survival than patients with tumors limited to the dermis (66% vs. 87%; *p* = 0.0135) [17].

Depth of invasion in BCC has been shown to vary according to histologic subtype and anatomic site, being the deepest when it comes to aggressive pattern subtypes in the head and neck, with a mean depth of invasion of 1.8 mm [28].

### Immunosuppression

Immunosuppression increases the risk for both developing NMSC and having a poorer outcome when diagnosed with NMSC [21, 29]. Nevertheless, immunosuppressed patients with NMSC share the same risk factors as the immunocompetent counterpart.

In a cohort of 205 patients treated for cSCC with surgery and adjuvant RT, immunosuppressed patients had dramatically poorer outcomes than immunocompetent patients with a progression-free survival of 38.7% vs. 71.6% at 2 years, respectively (*p* = 0.002) [30].

## Adjuvant RT for localized NMSC

Due to the lack of prospective studies validating the independent prognostication of the well-known risk factors, and also due to the heterogeneity of the available retrospective literature, it makes it that much more complex to clearly state consensually the role of adjuvant RT for localized NMSC (Table 1). A thorough knowledge of the albeit low quality level of literature, and a good amount of expertise in the field of NMSC, should make it possible to give treatment recommendations or even options, in the setting of a dedicated multidisciplinary tumor board.

In the context of surgically treated early stage BCC (i.e., T1 and T2, N0, M0) with positive or close margins, there are two options that must be discussed both at the multidisciplinary tumor board and with the patient. These options consist in observation, with close follow-up visits over at least a 5-year period, and adjuvant RT. The immediate addition of RT will confer a better local control, although one can argue that re-excision at the time of recurrence will yield the same survival outcome [14]. With a recurrence rate of 5% after a first excision, BCC local recurrence can be managed with a news surgical excision, but one must not neglect the increased recurrence rate after a second operation (14.7%), reaching 50% after a third and fourth surgical treatment [31]. Duinkerken et al. reported local control rates with adjuvant RT at 5 years of 92% for incompletely resected BCC, 90% for BCC treated at first recurrence, and 71% BCC treated at their second or more recurrence [32]. Moreover, aggressive BCC subtypes in the presence of inadequate margins are best not left undertreated, even more so when located in the mid-face area. Extensive salvage treatment with surgery and/or RT can result in unacceptable morbidity. Patients’ preference can play an important role in the final decision making, as factors such as fear of recurrence, or on the contrary fear of possible toxicity from more intensive treatment, or the impossibility to adhere to a tight long-term follow-up schedule, may outweigh physician’s preference.

As for resected early stage cSCC with close or positive margins, since the recurrence rate is significantly higher (up to 37%) compared with BCC (5%), and is also associated to regional and distant relapse, when immediate re-excision is not deemed feasible at the multidisciplinary tumor board, adjuvant RT should be offered in order to significantly reduce the local recurrence risk. Additionally RT should not be delayed, as a delay of more than 6 weeks after a suboptimal resection may impair the outcome [15]. As for the extent of RT volume, one must take into account tumor site and other risk factors (PNI, lymphatic invasion, vascular invasion), to eventually include the first echelons of the lymphatic drainage. Babington et al. observed more regional recurrences than local recurrence in patients treated with surgery for cSCC of the hair-baring lip (1/3 of patients with close/positive margins) [15], an anatomic site rich with lymphatic vessels.

A retrospective study on cPNI+ cSCC of the head and neck region treated with surgery and adjuvant RT, demonstrates that this patient population can achieve long-term survival with a 5-year disease-specific survival of 75% [33]. A neatly constructed treatment algorithm proposed by Jackson et al. in the presence of PNI clearly underlines the importance of a thorough pathology report [17]. All histopathological factors, such as detailed information on PNI presence, tumor differentiation, margins, size and so forth, should constitute the elements on which NMSC management currently should be based on. Based on their decisional algorithm, in the presence of cPNI, a combination of surgery, when feasible, and adjuvant RT, with curative or palliative intent, is advocated, whatever the histology. When pPNI is positive, for BCC adjuvant RT could become necessary in the presence of other risk factors, mainly a T2-T4 tumor, or a recurrent tumor; for cSCC, pPNI-specific high-risk factors, or the addition of any other histopathological risk factor confer a strong recommendation for adjuvant RT. pPNI-specific factors associated with poor prognosis include extratumoral PNI, involvement of nerves ≥ 0.1 mm, invasion beyond dermis, PNI in a recurrent tumor, extensive intratumoral spread [20].

Other scenarios in which adjuvant RT has shown to improve outcome include T3-T4 tumors, as well as recurrent cSCC [23, 25, 34, 35]. Of note, addition of concomitant chemotherapy using weekly radiosensitizing carboplatin to adjuvant RT for locally advanced resected T3-T4 tumors of the head and neck is not indicated based on the results of phase III TROG 05.01 trial [36••].

RT is contraindicated in genetic conditions predisposing to skin cancer (for example, basal cell nevus syndrome) and relatively contraindicated for patients with connective tissue diseases (for example, scleroderma and systemic lupus erythematous). Also, reirradiation should not be used in routine as a cumulative high dose increases the risk of complications to the underlying tissues [13•]. Finally, awareness should be made of the risk of RT induced secondary malignancies, especially when treating younger patients, for whom RT should not be withheld when the risk of local relapse is considered high by a panel of experts.

## Adjuvant radiotherapy techniques and doses

RT techniques used for the treatment of NMSC depend on multiple factors including primary tumor location, anatomical site, and tumor size. The most used RT techniques boil down to kilovoltage RT, electron beam RT, brachytherapy (low dose and high dose), and megavoltage photons by means of 3D conformal or intensity-modulated RT techniques (IMRT) [10••]. In general, kilovoltage RT allows treating small and superficial targets with maximum dose delivery to the skin surface, and a steady dose fall-off in the surrounding tissues; electron beams also are appropriate for small to medium size target volumes, with high surface dose and rapid dose fall-off; for correct coverage of extensive and more complex volumes, with deep extension and encountering tissues with different density, IMRT guaranties optimal dose distribution and allows also the delivery of different dose levels, for example when the nodal drainage must be treated with a lower so called “prophylactic” dose.

In the adjuvant setting, the most appropriate fractionation schedule must take into consideration both patient (age, performance status, patient preference) and tumor factors (site, primary tumor size, risk of nodal relapse). A total RT dose of 60–66 Gy with 2 Gy per fraction given daily, 5 times per week, over 6 weeks, is a normo-fractionated schedule recommended by the NCCN [6]. This fractionation is best adapted for extensive treatment volumes in fit patients. Other common fractionation schedules prescribed for NMSC are hypofractionated ones, meaning that the target receives higher doses per fraction, reducing the total number of fractions and also the overall treatment time. The total dose is biologically equivalent to the normo-fractionated 60 Gy. Usually elderly patient with small – medium treatment volumes benefit most from this approach. The NCCN recommends a very moderately hypofractionated schedule (50 Gy with 2.5 Gy per fraction, over 4 weeks) [6]. Alternative hypofractionated schedules are proposed by the ASTRO guidelines, delivering 15–17 daily fractions of 3Gy or more extreme schedules using fractions of 4 or 5 Gy delivered in 10 or 8 fractions, 2–4 times per week [10••].

Dundar et al. reviewed the effectiveness of four different RT schedules in 90 patients (140 tumor sites) with cSCC (76.6%) and BCC (15.5%) presenting various high-risk features such as PNI (*n* = 75), positive margins (*n* = 25), and recurrent disease (*n* = 114) [13•]. One group of patients received normo-fractionated RT with a mean dose of 60 Gy. The 3 other groups had hypofractionated RT delivered: 2.5 Gy per fraction up to a mean total dose of 50 Gy; 3–4 Gy per fraction for a total dose of 45 Gy; and 5–6 Gy per fraction for a total dose of 40 Gy. The two latter highly hypofractionated regimens were privileged for small volumes, locations with less cosmetic concern, and for very elderly patients with difficult treatment commutes. No difference in local control rates and toxicity were observed between the 4 fractionation groups. Therefore, the decision of which treatment regimen to use should take into account different elements, including the anatomic location, the tumor characteristics (shape, contour and deep) and proximity to normal tissues, but also patient preference and health-care related costs.

## Conclusions

Many adverse prognostic factors for loco-regional recurrence and distant metastasis in patients treated surgically for NMSC have been described in literature. However, the concomitant presence of multiple risk factors makes it harder to determine the contribution of each individual component. The indications for adjuvant RT in patients with localized resected NMSC harboring high-risk features remain debated, the data being from retrospective series.

Clinical trial or prospective registries adapted for both tumor and host risk factors are awaited to accurately determine the role of adjuvant RT on outcome of localized NMSC.
